# Comparison of clinical outcomes between luminal invasive ductal carcinoma and luminal invasive lobular carcinoma

**DOI:** 10.1186/s12885-016-2275-4

**Published:** 2016-03-25

**Authors:** Yayoi Adachi, Junko Ishiguro, Haruru Kotani, Tomoka Hisada, Mari Ichikawa, Naomi Gondo, Akiyo Yoshimura, Naoto Kondo, Masaya Hattori, Masataka Sawaki, Takashi Fujita, Toyone Kikumori, Yasushi Yatabe, Yasuhiro Kodera, Hiroji Iwata

**Affiliations:** Department of Breast Oncology, Aichi Cancer Center Hospital, 1-1, Kanokoden, Chikusaku, Nagoya, 464-8681 Japan; Department of Transplantation and Endocrine Surgery, Nagoya University Graduate School of Medicine, 65 Tsurumai, Showaku, Nagoya, 466-8560 Japan; Department of Pathology and Molecular Diagnostics, Aichi Cancer Center Hospital, 1-1, Kanokoden, Chikusaku, Nagoya, 464-8681 Japan; Department of Gastroenterological Surgery, Nagoya University Graduate School of Medicine, 65 Tsurumai, Showaku, Nagoya, Aichi 466-8560 Japan

**Keywords:** Invasive lobular carcinoma, Invasive ductal carcinoma, Luminal, Prognosis

## Abstract

**Background:**

The pathological and clinical features of invasive lobular carcinoma (ILC) differ from those of invasive ductal carcinoma (IDC). Several studies have indicated that patients with ILC have a better prognosis than those with ductal carcinoma. However, no previous study has considered the molecular subtypes and histological subtypes of ILC. We compared prognosis between IDC and classical, luminal type ILC and developed prognostic factors for early breast cancer patients with classical luminal ILC.

**Methods:**

Four thousand one hundred ten breast cancer patients were treated at the Aichi Cancer Center Hospital from 2003 to 2012. We identified 1,661 cases with luminal IDC and 105 cases with luminal classical ILC. We examined baseline characteristics, clinical outcomes, and prognostic factors of luminal ILC.

**Results:**

The prognosis of luminal ILC was significantly worse than that of luminal IDC. The rates of 5-year disease free survival (DFS) were 91.9 % and 88.4 % for patients with luminal IDC and luminal ILC, respectively (*P* = 0.008). The rates of 5-year overall survival (OS) were 97.6 % and 93.1 % for patients with luminal IDC and luminal ILC respectively (*P* = 0.030). Although we analyzed prognosis according to stratification by tumor size, luminal ILC tended to have worse DFS than luminal IDC in the large tumor group. In addition, although our analysis was performed according to matching lymph node status, luminal ILC had a significantly worse DFS and OS than luminal IDC in node-positive patients. Survival curves showed that the prognosis for ILC became worse than IDC over time. Multivariate analysis showed that ILC was an important factor related to higher risk of recurrence of luminal type breast cancer, even when tumor size, lymph node status and histological grade were considered.

**Conclusions:**

Luminal ILC had worse outcomes than luminal IDC. Consequently, different treatment approaches should be used for luminal ILC than for luminal IDC.

**Electronic supplementary material:**

The online version of this article (doi:10.1186/s12885-016-2275-4) contains supplementary material, which is available to authorized users.

## Background

Invasive lobular carcinoma (ILC) constitutes 5 % or less of the cases of breast carcinoma in most series [1]. However, the frequency of ILC has been reported to be as high as 10–14 % of invasive carcinomas according to less restrictive diagnostic criteria [1]. The pathological and clinical features of ILC differ from those of invasive ductal carcinoma (IDC) [2–4]. The overall 10-year survival of patients with ILC is higher than patients with IDC [1]. The typical pathological feature of ILC are lack of cohesion among tumor cells and the presence of slender strands of cells arranged in a linear fashion [1, 5].

Further, ILC can be discriminated between classical and pleomorphic forms. Classical ILC consists of small, uniform cells with round nuclei and inconspicuous nucleoli. Pleomorphic ILC consists of cells larger than those in classical ILC with relatively abundant, eosinophilic cytoplasm. Classical ILC has a more favorable prognosis than the pleomorphic form [4].

In invasive ductal carcinoma, the prognosis differs widely according to molecular subtype. Unfortunately, there is no evidence of any difference in prognosis between ILC and IDC with similar molecular subtypes. The aims of this study were to compare prognosis between IDC and ILC of the luminal type and to develop prognostic factors for early breast cancer patients with classical ILC.

## Methods

### Study population

Four thousand one hundred ten breast cancer patients underwent surgery at the Aichi Cancer Center Hospital from 2003 to 2012. We obtained the clinical and pathological data from patient’s records retrospectively. The diagnosis of ILC was defined by a typical appearance of microscopic pathological features and immunohistochemical staining of E-Cadherin. Variants of ILC were excluded.

Patients for whom information on estrogen receptor (ER), progesterone receptor (PgR) and human growth factor receptor 2 (HER2) status was unavailable were excluded from this study. Furthermore, patients with cT4 breast cancer, metastasis at presentation, bilateral breast cancer, a history of other cancer, or neo adjuvant therapy were also excluded.

Our study was approved by the Institutional Review Board of Aichi Cancer Center Hospital. Informed consent was obtained from each patient in oral and written form before inclusion in the study.

### Pathological assessment and definition of molecular subtypes

Histopathological diagnoses of ILC and IDC using hematoxylin–eosin staining were made by several pathologists at Aichi Cancer Center Hospital. Hormone receptor (ER and PgR) status was determined by immunohistochemical staining. Hormone receptor-positive status was defined as a score of equal or greater than 3 of ER on the Allred Score [6]. HER2 positive was defined as a Herceptest-score of 3+ or fluorescent in situ hybridization (FISH) positive following a Herceptest-score of 2 + [7]. The definition of luminal type was determined as ER positive and HER2 negative. Histological grading was performed using the Nottingham histological grading system. Tumor stage was stratified according to the AJCC 7th edition TNM staging system for breast cancer. The dataset supporting the conclusions of this article is included within the article and its additional file (Additional file 1).

### Statistical analysis

Differences in clinicopathological features between IDC and ILC were compared using chi-squared analysis and Fisher’s test. The log-rank test and estimation of hazard ratios using COX regression analysis were used for univariate analysis, and cumulative survival curves were derived by Kaplan-Meier methods. Disease free survival (DFS) was defined as the time from the date of operation to relapse including local recurrence, or death. Overall survival (OS) was defined as the time from the date of operation to death from any cause. Cox regression analysis using proportional hazards modeling was used in multivariate analyses. The proportional hazards assumption was verified using the Schoenfeld Residuals Test. All tests were two-sided, and a *P* value of <0.05 was considered statistically significant. All data were analyzed using STATA software version12.0.

## Results

### Patient and tumor characteristics

We identified 1,998 cases of IDC and 115 cases of ILC after excluding patients according to the criteria described above as well as cases of carcinoma *in situ*. Among these, the number of cases of IDC and ILC with luminal subtype were 1,661(83 % of IDC) and 104(90 % of ILC), respectively. Among ILC, 3 % were hormone receptor positive and HER2 positive and 2 % were hormone receptor negative and HER2 positive, and 5 % were triple negative. The median follow-up time was 64 months (0–126). The clinical and pathological tumor characteristics of luminal IDC and luminal ILC are shown in Table 1. The tumor size of luminal ILC was larger than that of luminal IDC (*P* = 0.002). Luminal ILC was more likely to have a lower histological grade than luminal IDC (*P* < 0.001).

**Table 1:** Patient characteristics

	Luminal IDC (n = 1661)		Luminal ILC (n = 104) (n = 104)		
	n	%	n	%	*P*-value
Median follow-up time(months)	53		49.5		
Age(years)					
Median	53		53		
< 50	678	40	44	42	
≧50	982	59	60	57	0.768
Menopause status					
Pre	812	48	52	50	
Post	844	50	50	48	0.703
Histological grade					
1	486	29	68	65	
2	875	52	26	25	
3	248	14	2	1	<0.001
Tumor size					
T1	1174	70	57	54	
T2	422	25	40	38	
T3	63	3	7	6	0.002
ER(Allred score)					
3	19	1	0	0	
4	31	2	0	0	
5	30	2	1	1	
6	62	4	5	5	
7	246	15	15	14	
8	1273	76	83	80	0.575
HER2 status					
0	439	26	25	24	
1	995	60	67	64	
2(FISH-)	227	14	12	12	0.682
Lymph node status					
Positive	489	29	34	32	
Negative	1021	61	61	58	0.492
Initial surgical treatment					
BCS	779	47	41	39	
Mastectomy	882	53	63	61	0.007
Positive margins	38	2.2	7	6.7	0.016
Endocrine therapy					
Yes	1424	85	99	95	
No	231	13	5	4	0.008
Chemotherapy					
Yes	672	40	44	42	
No	982	59	60	57	0.735

*IDC* invasive ductal carcinoma, *ILC* invasive lobular carcinoma, *FISH* fluorescent in situ hybridization, *BCS* breast conserving surgery

Positive margins were more frequently found in luminal ILC than in luminal IDC (*P* = 0.016). The majority of patients with luminal ILC were treated with adjuvant hormonal therapy. In addition, those with luminal ILC were more likely to receive adjuvant hormonal therapy than those with luminal IDC (*P* = 0.008). However, there were no significant differences in other characteristics (age, menopausal status, lymph node status and chemotherapy) between the two groups.

### Univariate analysis of luminal type

**Fig. 1:**
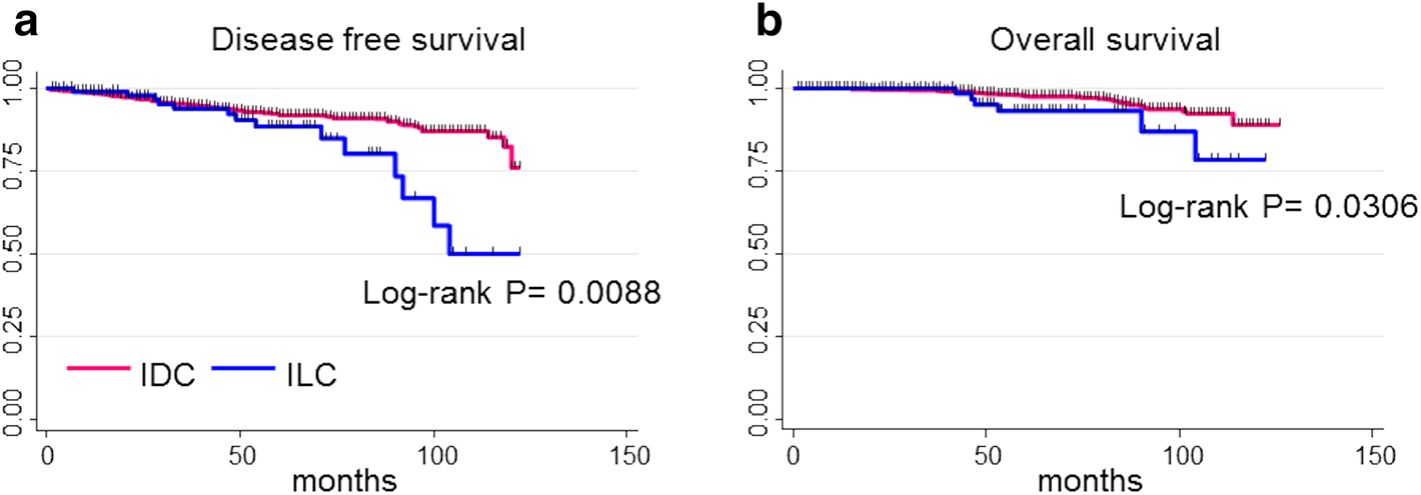
Patient outcomes of luminal IDC and luminal ILC; (**a**) disease-free survival (**b**) overall survival, *IDC* invasive ductal carcinoma, *ILC* invasive lobular carcinoma

The prognosis of luminal ILC was significantly worse than that of luminal IDC. The 5-year DFS was 91.9 % and 88.4 % for patients with luminal IDC and luminal ILC, respectively (*P* = 0.008), while the 5-year OS was 97.6 % and 93.1 %, respectively, for patients with luminal IDC and luminal ILC (*P* = 0.030) (Fig. 1).

**Fig. 2:**
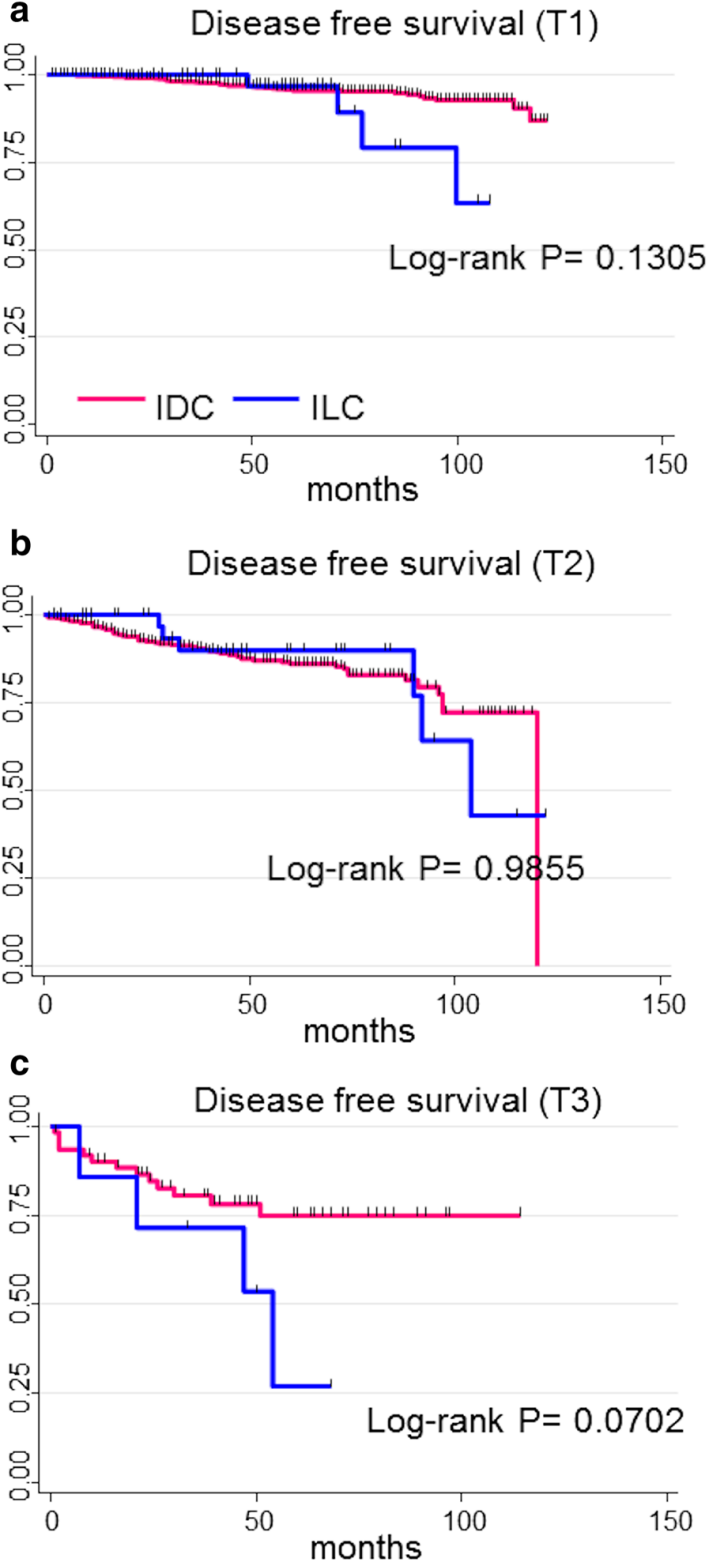
Patient outcomes of luminal IDC and luminal ILC stratified according to tumor size; (**a**) T1 (**b**) T2 (**c**) T3, *IDC* invasive ductal carcinoma, *ILC* invasive lobular carcinoma

**Fig. 3:**
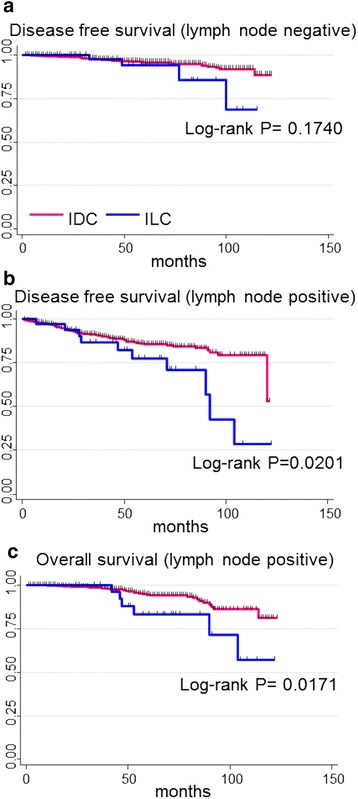
Patient outcomes of luminal IDC and luminal ILC stratified according to lymph node status; (**a**) disease free survival in lymph node-negative patients (**b**) disease free survival in lymph node-positive patients (**c**) overall survival in lymph node-positive patients, *IDC* invasive ductal carcinoma, *ILC* invasive lobular carcinoma

The survival curves for luminal IDC and luminal ILC after stratification by tumor size are shown in Fig. 2. There were no significant differences in DFS between the two groups (Fig. 2) However, the 5-year DFS of luminal ILC tended to be worse than that of luminal IDC in cases with large tumors (T3 cases) (26.7 % vs 74.9 %, *P* = 0.07) (Fig. 2c). The survival curves for luminal IDC and luminal ILC after stratification by lymph node status are shown in Fig. 3. There were no significant differences in DFS between the two groups in the node negative population (Fig. 3a). However, the 5-year DFS of luminal ILC was significantly worse than that of luminal IDC in the node positive population (77.4 % vs 85.5 %, *P* = 0.02) (Fig. 3b). Furthermore, the 5-year OS of luminal ILC was also significantly worse than that of luminal IDC in the node-positive population (83.3 % vs 94.4 %, *P* = 0.017) (Fig. 3c).

**Fig. 4:**
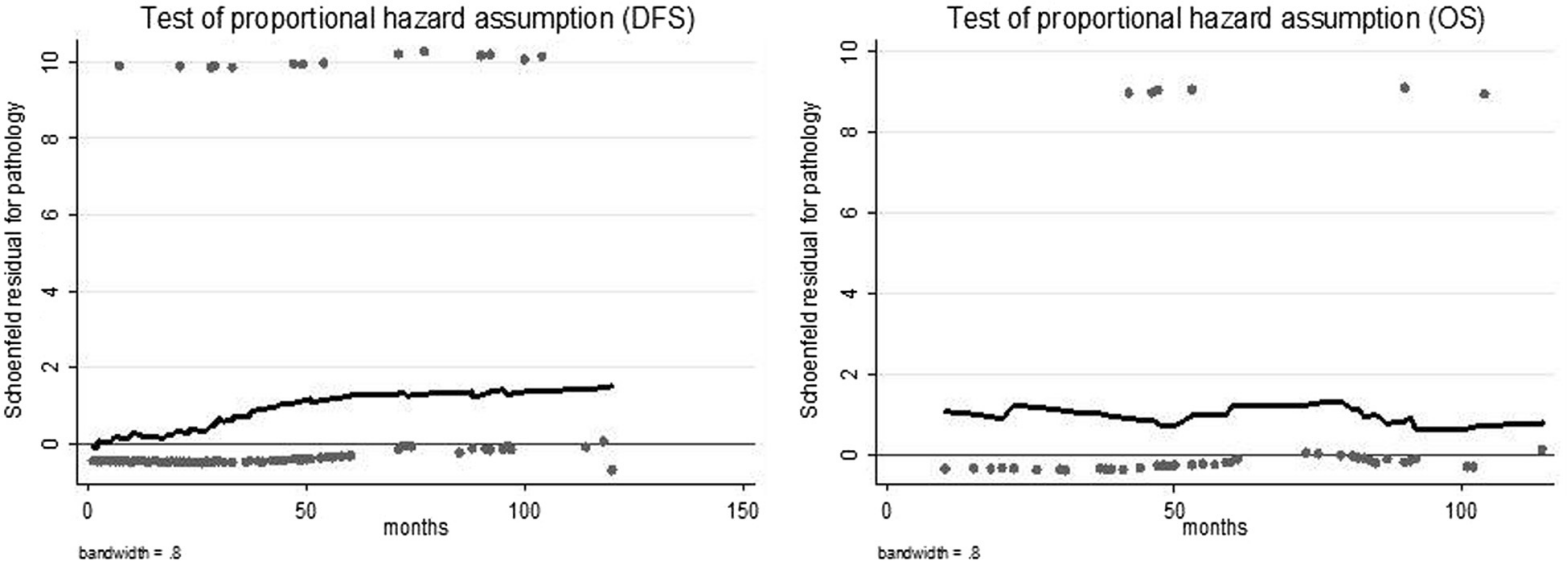
Assessment of the nature of non-proportional hazards when patients’ with ILC are opposed to IDC using the Schoenfeld Residuals Test; *ILC* invasive lobular carcinoma, *IDC* invasive ductal carcinoma, *DFS* disease free survival, *OS* overall survival

In univariate analysis, ILC (*P* = 0.008), large tumor size (*P* < 0.001), lymph node positivity (*P* < 0.001), and high grade (*P* < 0.001) were worse prognostic factors for luminal type breast cancer (Table 2). The test of non-proportional hazards for DFS for the variable ‘pathology type’, using the Schoenfeld Residuals Test, was insignificant. Time split analysis and graphical results suggested the presence of an association between pathology type and DFS over time (Table 3, Fig. 4).

**Table 2:** Univariate analysis for luminal types (ILC and IDC)

	DFS		OS	
Variables	HR (95 % CI)	*P*-value	HR (95 % CI)	*P*-value
Age				
≧50	1.0		1.0	
< 50	1.14(0.80–1.64)	0.455	1.17(0.65–2.11)	0.591
Menopause status				
Post	1.0		1.0	
Pre	1.06(0.75–1.51)	0.708	1.20(0.68–2.11)	0.513
Tumor size				
< 2	1.0		1.0	
2 ≤ T < 5	3.82(2.62–5.58)		6.08(3.19–11.57)	
5≥	8.24(4.72–14.39)	<0.001	11.81(4.74–29.42)	<0.001
Lymph node status				
Negative	1.0		1.0	
Positive	3.51(2.40–5.12)	<0.001	6.26(3.17–12.36)	<0.001
Pathology type(ILC,IDC)				
IDC	1.0		1.0	
ILC	2.06(1.18–3.60)	0.008	2.48(1.05–5.84)	0.030
Histological grade				
1	1.0		1.0	
2	1.98(1.19–3.31)		2.03(0.82–5.00)	
3	4.41(2.53–7.70)	<0.001	5.52(2.15–14.12)	<0.001

*IDC* invasive ductal carcinoma, *ILC* invasive lobular carcinoma, *DFS* disease free survival, *OS* overall survival, *HR* hazard ratio, *95 % CI* 95 % confidence interval

**Table 3:** Univariate analysis for luminal types (ILC and IDC, analysis of the split times)

	0–5 years of follow-up		5 years to end of follow-up	
Variable: pathology type	HR (95 % CI)	*P*-value	HR (95 % CI)	*P*-value
DFS				
IDC	1.0		1.0	
ILC	1.32(0.64–2.72)	0.440	7.42(2.94–18.74)	<0.001
OS				
IDC	1.0		1.0	
ILC	2.74(0.95–7.91)	0.051	2.09(0.48–9.01)	0.308

*IDC* invasive ductal carcinoma, *ILC* invasive lobular carcinoma, *DFS* disease free survival, *OS* overall survival, *HR* hazard ratio, *95 % CI* 95 % confidence interval

### Multivariate analysis of luminal type

Multivariate analysis was performed using Cox regression models to determine the independent prognostic factors of luminal type breast cancer. Factors in this analysis were pathological type (IDC or ILC), age, tumor size, lymph node status, histological grade, endocrine therapy, and chemotherapy. Pathological type, tumor size, lymph node status, histological grade, and endocrine therapy were prognostic factors independently associated with recurrence of luminal type breast cancer (Table 4). Moreover, tumor size and lymph node status were the prognostic factors for better survival in luminal type breast cancer.

**Table 4:** Multivariate analysis for luminal types (IDC, ILC)

	DFS		OS	
Variables	HR (95 % CI)	*P*-value	HR (95 % CI)	*P*-value
Age				
≧50	1.0		1.0	
< 50	1.32(0.88–1.99)	0.176	1.60(0.79–3.25)	0.184
Tumor size				
< 2	1.0		1.0	
2 ≤ T < 5	2.35(1.49–3.71)		3.85(1.79–8.26)	
5≥	4.04(2.11–7.73)	<0.001	5.84(1.65–14.15)	<0.001
Lymph node status				
Negative	1.0		1.0	
Positive	2.28(1.37–3.79)	0.001	3.06(1.39–8.15)	0.010
Pathology type				
IDC	1.0		1.0	
ILC	2.49(1.28–4.85)	0.009	1.90(0.63–5.74)	0.262
Histological grade				
1	1.0		1.0	
2	1.77(1.01–3.10)		1.51(0.58–3.91)	
3	2.31(1.22–4.38)	0.009	2.44(0.87–6.81)	0.060
Endocrine therapy				
No	1.0		1.0	
Yes	0.40(0.25–0.70)	0.002	0.35(0.15–0.82)	0.017
Chemotherapy				
No	1.0		1.0	
Yes	1.29(0.74–2.25)	0.320	1.02(0.42–2.50)	0.745

*IDC* invasive ductal carcinoma, *ILC* invasive lobular carcinoma *DFS* disease free survival, *OS* overall survival, *HR* hazard ratio, *95%CI* 95 % confidence interval

### Univariate analysis and multivariate analysis in luminal ILC

In univariate analysis, large tumor size (*P* < 0.001) and lymph node positivity (*P* = 0.015) correlated with significantly worse DFS in luminal ILC (Table 4). When tumor size and lymph node status were entered in a multivariate analysis for luminal ILC recurrence, large size was an independent prognostic factor (*P* = 0.024) (Table 5).

**Table 5:** Univariate analysis and multivariate analysis for recurrence of luminal ILC

	DFS	
Variables	HR(95%CI)	*P*-value
Univariate analysis		
Age		
≧50	1.0	
< 50	0.82(0.28–2.38)	0.717
Menopause status		
Post	1.0	
Pre	0.81(0.27–2.38)	0.707
Tumor size		
< 2	1.0	
2 ≤ T < 5	1.84(0.51–6.60)	
5≥	19.53(4.11–92.82)	<0.001
Lymph node status		
Negative	1.0	
Positive	3.78(1.18–12.07)	0.015
Histological grade		
1	1.0	
2	2.25(0.64–7.89)	
3	2.20(0.24–20.04)	0.400
Multivariate analysis		
Tumor size		
< 2	1.0	
2 ≤ T < 5	1.49(0.39–5.61)	
5≥	10.18(1.79–57.75)	0.024
Lymph node status		
Negative	1.0	
Positive	2.18(0.58–8.14)	0.207

*ILC* invasive lobular carcinoma, *DFS* disease free survival, *HR* hazard ratio, 95 % *CI* 95 % confidence interval

## Discussion

ILC is the second most common type of invasive breast cancer. The clinical and biological characteristics of ILC differ from those of IDC [2–4]. Several studies have indicated that patients with ILC have a better prognosis than patients with ductal carcinoma. Currently, breast cancer can be classified into four molecular subtypes (luminal A, luminal B, HER2-positive, or triple negative) based on their expression of hormone receptors, HER2, and Ki-67. The subtypes of luminal, HER2-positive, and triple negative in this study are defined as ER positive and HER2 positive or negative, ER negative and HER2 positive, ER negative and HER2 negative, respectively [8–10]. Although there are many reports that these molecular subtypes are strongly associated with prognosis in IDC [8, 9], there are few reports of any association in ILC. Iorfida et al*.* showed that each molecular subtype had different outcomes in ILC, as they do in IDC [4]. They reported that ILC was more likely to be associated with luminal type than IDC, while luminal A had higher rates of DFS and OS than other molecular subtypes of ILC. However, they did not compare prognosis between IDC and ILC stratified into molecular subtypes.

Moreover, ILC is classified into histological subtypes (classical, alveolar, solid, tubulolobular, pleomorphic, and mixed type). Each histological subtype has a different prognosis [1, 5]. Among them, pleomorphic ILC has a worse prognosis than classical ILC [4]. However, there has been no previous study in which molecular subtypes and histological subtypes of ILC were considered. Therefore, we performed a retrospective analysis to compare the prognosis between luminal IDC and classical luminal ILC.

In this study, luminal ILC patients had larger tumors than luminal IDC patients. Moreover, luminal ILC tumors were of a lower grade than luminal IDC tumors. The characteristics of these patients tended to be similar to those previously reported for all types of ILC [2, 4, 11–15]. The larger size of ILC can be attributed to the biological behavior of ILC. Their indolent infiltration into stroma without a desmoplastic reaction could make it difficult to detect small ILC on radiological examination [11, 12]. Although previous studies have shown that the rate of lymph node positivity is higher in ILC [3, 12, 15], there was no difference in lymph node metastasis between the two groups in this study. We believe that this may have been due to the exclusion of pleomorphic ILC with aggressive clinical features.

The number of patients with luminal IDC who received hormone therapy was significantly lower than those with luminal ILC in this study. We consider that the reason for this difference may be as follows. There was a significant difference in tumor size between luminal IDC and luminal ILC. Luminal IDC patients who did not receive hormone therapy had very small tumors (mainly less than 10 mm).

In our study, the prognosis of luminal ILC was significantly worse than that of luminal IDC. Although we analyzed prognosis according to stratification by tumor size, luminal ILC tended to have worse DFS than luminal IDC in the large tumor group. In addition, although our analysis was performed according to matching lymph node status, luminal ILC had a significantly worse DFS and OS than luminal IDC in node-positive patients. DiCostanzo et al. compared IDC and classical ILC, matched for age, tumor size and nodal status [16]. They showed that classical ILC had better DFS than IDC. The difference between our results and theirs might be accounted for by the fact that they did not consider molecular subtypes. The large study by Wasif et al*.* also reported that stage-matched prognosis was better for ILC than IDC [3]. They reported that ILC was more often ER positive and suggested that the favorable prognosis of ILC might be related to high expression of ER.

In our study, multivariate analysis showed that ILC was an important factor related to higher risk of recurrence of luminal type breast cancer, even when tumor size, lymph node status and histological grade were considered. Tubiana-Hulin et al*.* reported that pathological type (IDC/ILC) was not related to DFS or OS in their multivariate analysis [15]. However, their study was not limited to luminal type breast cancer. To date, this study is the first attempt to compare the prognosis of luminal IDC and luminal ILC. In addition, this study indicates that ILC is an important prognostic factor for luminal type breast cancer.

In our study, the most important prognostic factor for luminal ILC was tumor size. This result was basically the same as that reported from previous studies, such as the finding that tumor size and lymph node status were prognostic factors for ILC, as reported for IDC [3, 4, 13, 16].

The results of our study might be related to responsiveness to adjuvant therapies. In advanced cases such as those with large tumors or which are lymph node-positive, adjuvant chemotherapy was generally performed. In fact, in this study, most T3 and lymph node-positive patients were administered adjuvant chemotherapy (luminal ILC: 85 % of T3, 97 % of LN+, luminal LDC: 87 % of T3, 80 % of LN+). However, the response of ILC to primary chemotherapy was significantly lower than that of IDC reported in a previous study [14, 15, 17]. Therefore, in advanced cases that usually receive adjuvant chemotherapy, patients with luminal ILC might show worse prognosis than those with luminal IDC. Regarding hormone therapy, a previous study reported that poorer DFS was observed for ILC patients with endocrine-responsive tumors who did not receive any adjuvant hormonal therapy [12, 15], and hormonal therapy might be considered to improve the outcome. There have been several large studies which reported that the prognosis for ILC became worse than IDC over time [2, 12] and similar patterns were observed in our results. A time dependent association between pathology type and DFS was observed. We found an increased hazard for DFS among patients with ILC. These results imply that ILC exhibits indolent but progressive clinical behavior. This might also be an important factor when considering the treatment options for ILC. Some authors have considered that extended adjuvant hormone therapy might be necessary for luminal ILC [4]. Several studies have shown that the metastatic patterns of ILC differ from those of IDC [12, 13]. Although we did not take into consideration the site and timing of metastasis in this study, this difference might also be related to the worse prognosis of ILC. Moreover, there were more cases with positive margins in luminal ILC than in luminal IDC in this study. It could be due to indistinct margins of ILC in imaging study and therefore, related to higher likelihood of local recurrences in luminal ILC than in luminal IDC.

One limitation of this study is that our results were based on a retrospective analysis. HER2 status could be inconsistent during the study period due to several changes of the definition of HER2 positivity [7]. Moreover, sufficient data of PgR and Ki-67 required to distinguish between luminal A and luminal B were not available, therefore we defined luminal type as ER positive and HER2 negative in this study and we could not discuss about a difference between luminal A ILC and luminal B ILC. However, Engtrom et al*.* reported that ILC had worse prognosis than IDC for both luminal A and luminal B. Additionally, they showed that luminal A ILC and luminal B ILC had similar prognosis [18]. Even though we used different definitions of ‘luminal’, we had same finding that luminal ILC had worse prognosis than luminal IDC. This might mean that PgR and Ki-67 are not associated with the prognosis of luminal ILC.

Despite some limitations, this is the first study which suggests that ILC is an independent prognostic factor for luminal type breast cancer, and the results suggest that it may be necessary to reconsider the clinical approach for luminal ILC. In order to examine this hypothesis, several gene-expression profiling studies will be required to determine whether ILC has different patterns of gene expression from IDC even if histological grade and molecular subtypes are matched [19]. Therefore, other scientific approaches such as gene-expression profiling studies may provide answers to the questions raised about clinical behavior and systemic approaches to ILC.

## Conclusions

In conclusion, luminal ILC was associated with worse outcomes than luminal IDC. Consequently, luminal ILC should be approached with a different adjuvant therapy from luminal IDC, and a prospective clinical trial of adjuvant therapies for luminal ILC is required. Other approaches such as genomics are also essential to answer the question of clinical behavior and to identify appropriate therapies for ILC.

## Additional file

Additional file 1: Dataset supporting the conclusions of this article. (XLSX 94 kb)

## Abbreviations

BCS: breast conserving surgery
DFS: disease free survival
ER: estrogen receptor
FISH: fluorescent in situ hybridization
HER2: human epidermal growth factor receptor2
IDC: invasive ductal carcinoma
ILC: invasive lobular carcinoma
OS: overall survival
PgR: progesterone receptor
